# Complete Genome Sequence of *Bifidobacterium longum* 105-A, a Strain with High Transformation Efficiency

**DOI:** 10.1128/genomeA.01311-14

**Published:** 2014-12-18

**Authors:** Yu Kanesaki, Hisayoshi Masutani, Mikiyasu Sakanaka, Yuh Shiwa, Takatomo Fujisawa, Yasukazu Nakamura, Atsushi Yokota, Satoru Fukiya, Tohru Suzuki, Hirofumi Yoshikawa

**Affiliations:** a Genome Research Center, Tokyo University of Agriculture, Setagaya, Tokyo, Japan; b Graduate School of Applied Biological Sciences, Gifu University, Gifu, Gifu, Japan; c Laboratory of Microbial Physiology, Research Faculty of Agriculture, Hokkaido University, Sapporo, Hokkaido, Japan; d Center for Information Biology, National Institute of Genetics, Research Organization of Information and Systems, Mishima, Shizuoka, Japan; e the United Graduate School of Agricultural Science, Gifu University, Gifu, Gifu, Japan; f Department of Bioscience, Tokyo University of Agriculture, Setagaya, Tokyo, Japan

## Abstract

*Bifidobacterium longum* 105-A shows high transformation efficiency and allows for the generation of gene knockout mutants through homologous recombination. Here, we report the complete genome sequence of strain 105-A. Genes encoding at least four putative restriction-modification systems were found in this genome, which might contribute to its transformation efficiency.

## GENOME ANNOUNCEMENT

Bifidobacteria are naturally found in the human large intestine and are used as probiotic bacteria due to their beneficial effects on human health (1). Bifidobacteria are known for their difficulties with gene manipulation, especially in the generation of gene knockout mutants (2–4). This is mainly caused by the low transformation efficiencies demonstrated by *Bifidobacterium* strains, in part due to their restriction-modification (R-M) systems (2–6). Among the bifidobacteria, *Bifidobacterium longum* 105-A (7), isolated from human feces, has shown exceptionally high transformation efficiency (approximately 10^4^ to 10^6^ transformants/μg DNA) with several plasmid DNAs (6–9). Moreover, gene knockout mutants of *B. longum* 105-A and its derivative strain have been successfully generated using homologous recombination systems (8, 10–12). Thus, strain 105-A has become a representative host strain for functional genomics studies of bifidobacteria. Here, we deciphered the complete genome sequence of *B. longum* 105-A.

Genomic DNA was isolated from *B. longum* 105-A as described previously (8) and sequenced through the massively parallel sequencing method using a PacBio RS II (Pacific Biosciences of California, Inc., Menlo Park, CA, USA) and the Genome Analyzer IIx (GAIIx; Illumina, Inc., San Diego, CA, USA). Approximately 740- and 253-fold sequence coverages were obtained by the PacBio RS II and GAIIx, respectively. Sequence reads from the PacBio RS II and GAIIx were assembled using Celera Assembler version 7.0 and Velvet 0.7.55, respectively. The Celera Assembler generated short contigs and a super contig with a gap region. The gap was covered by a combination of two contigs assembled from the GAIIx data and a short contig assembled from the PacBio RS II data. The junction regions among the contigs were confirmed by Sanger sequencing. Error correction of the homopolymer region was performed by mapping the short read data obtained by the GAIIx.

The circular chromosome of *B. longum* 105-A contains 2,290,145 bp, for which 1,878 open reading frames (ORFs), 56 tRNA genes, 1 transfer-messenger RNA (tmRNA) gene, and 4 rRNA operons were predicted by g-MiGAP (13). The average G+C content of the genome is 60.06%. The total number of ORFs in the *B. longum* 105-A genome is higher than that in the representative *B. longum* strain, NCC2705 (14), which has 1,728 ORFs, 57 tRNA genes, 1 tmRNA gene, and 4 rRNA operons. In addition, local BLASTx analysis using REBASE entries (15) revealed that the *B. longum* 105-A genome contains genes encoding at least four types of putative R-M systems: a type I system comprising BL105A_1442 (a methyltransferase), BL105A_1441 (a specificity subunit), and BL105A_1439 (an endonuclease); one type II system comprising BL105A_1060 (an endonuclease) and BL105A_1059 (a methyltransferase); another type II system comprising BL105A_0366 (an endonuclease/methyltransferase); and a type IV system comprising BL105A_0073 (an endonuclease). Further functional analysis of the R-M system genes will clarify their contributions to transformation efficiency. The complete genome sequence of *B. longum* 105-A will largely contribute to postgenomic or functional genomics studies of bifidobacteria.

### Nucleotide sequence accession number.

The complete genome sequence of *B. longum* 105-A has been deposited in the DDBJ/EMBL/GenBank database under accession no. AP014658.
